# Myeloid‐Derived LGALS9‐P4HB Immune Interaction Promotes Metastasis in Gastric Cancer Through Enhanced Cell Proliferation and Lipid Metabolism

**DOI:** 10.1111/jcmm.70661

**Published:** 2025-06-18

**Authors:** Xiaobin Zhu, Yating Zhang, Aiping Yu, Xiao Xiao

**Affiliations:** ^1^ Department of Spine Surgery and Musculoskeletal Tumor Zhongnan Hospital of Wuhan University Wuhan Hubei Province People's Republic of China; ^2^ Institute of Health Inspection and Testing, Hubei Provincial Center for Disease Control and Prevention Wuhan Hubei China; ^3^ Department of Infectious Disease Prevention and Control Dongxihu Centers for Disease Prevention and Control Wuhan Hubei China; ^4^ Department of Laboratory Medicine Zhongnan Hospital of Wuhan University Wuhan China

**Keywords:** cellular communication, gastric cancer, LGALS9, lipid metabolism, metastasis, P4HB, tumour microenvironment

## Abstract

Metastasis remains the primary cause of mortality in gastric cancer patients; however, the underlying mechanisms driving this process remain incompletely understood. Here, we performed an integrated single‐cell analysis of gastric cancer primary tumours and their corresponding liver and lymph node metastases to identify critical intercellular communication networks driving the metastatic process. Notably, gene expression analysis of metastatic tissues showed significant upregulation of cholesterol metabolism and PPAR signalling pathway (a nuclear receptor–mediated regulatory system that orchestrates lipid metabolism, adipogenesis and energy homeostasis) genes compared to primary tumours. Our analysis revealed that myeloid cell–derived Galectin‐9 (LGALS9) and its receptor beta‐subunit of prolyl 4‐hydroxylase (P4HB) on epithelial cells constitute a previously uncharacterized ligand–receptor interaction involved in gastric cancer metastasis. Functional experiments confirmed that the activation of P4HB by LGALS9 significantly enhanced proliferation, epithelial‐mesenchymal transition (EMT) and lipid metabolism in gastric cancer cells, while pharmacological inhibition of P4HB reversed these effects. Collectively, our findings establish the myeloid‐derived LGALS9‐P4HB interaction as a crucial mediator of gastric cancer metastatic colonisation through modulation of lipid metabolism, suggesting a potential therapeutic target for metastatic gastric cancer.

## 1 Introduction

Gastric cancer represents the fifth most common malignancy and the third leading cause of cancer‐related deaths worldwide, with an estimated 783,000 deaths annually [1, 2, 3]. Despite advances in multimodal therapies, the 5‐year survival rate remains dismal at approximately 30%, largely attributed to the high incidence of metastasis at diagnosis [4]. Metastasis, a complex multistep process involving detachment from the primary tumour, invasion, circulation, extravasation and colonisation of distant organs, represents the principal determinant of poor prognosis in gastric cancer patients [5, 6].

The success of metastatic colonisation heavily depends on the intricate interplay between tumour cells and the surrounding microenvironment at the secondary site. Recent findings suggest that tumour cells must adapt to the unique microenvironmental pressures of the metastatic niche to survive and proliferate [7]. This adaptation process involves extensive cellular communication between metastatic cells and resident stromal and immune cells [8, 9]. However, the specific cellular interactions and molecular pathways that facilitate metastatic colonisation in gastric cancer remain largely uncharacterised.

Recent technological advances in single‐cell RNA sequencing (scRNA‐seq) have provided unprecedented opportunities to dissect the heterogeneity of tumours and their microenvironment with cellular resolution [10, 11]. This approach enables comprehensive profiling of diverse cell types, states and their intercellular communications within complex tissues. By applying scRNA‐seq to primary tumours and corresponding metastases, researchers can identify cell‐specific alterations and intercellular signalling networks that drive the metastatic cascade.

Galectins, a family of β‐galactoside‐binding proteins, have emerged as critical regulators of cancer progression and metastasis [12, 13, 14]. Among them, Galectin‐9 (LGALS9) has been implicated in various aspects of cancer biology, including immune evasion, angiogenesis and cell adhesion [15, 16]. However, its role in gastric cancer metastasis and the identity of its key interacting partners in this context remain elusive.

Protein disulfide isomerase (P4HB), primarily known for its role in protein folding in the endoplasmic reticulum, has recently been recognised as a cell surface receptor involved in diverse cellular processes [17]. Emerging evidence suggests an elevated P4HB expression in various cancers and its association with poor prognosis [18], yet its function in gastric cancer metastasis remains unexplored.

In this study, we performed an integrated scRNA‐seq analysis of primary gastric cancer tumours and their corresponding liver and lymph node metastases to comprehensively profile the cellular composition and intercellular communication networks in the metastatic microenvironment. We identified a novel interaction between myeloid cell–derived LGALS9 and epithelial cell–expressed P4HB that promotes metastatic colonisation through enhanced proliferation, epithelial‐mesenchymal transition (EMT) and altered lipid metabolism. Pharmacological inhibition of P4HB significantly attenuated these pro‐metastatic effects, suggesting a potential therapeutic strategy for metastatic gastric cancer.

## 2 Materials and Methods

### 2.1 Single‐Cell Data Processing and Integration

Single‐cell RNA sequencing data from primary gastric cancer tumours (GC) and matched liver metastases (LM) and lymph node metastases (LymphM) were obtained from the Gene Expression Omnibus (GEO) under accession numbers GSE246662 [10] and GSE163558 [11]. In total, data from 43,720 GC cells, 36,419 LM cells and 12,086 LymphM cells were analysed. Raw expression matrices were processed using the Seurat v5 package in R. Quality control was performed by filtering cells with fewer than 200 genes, more than 6000 genes, or more than 10% mitochondrial gene content. Data normalisation was performed using the SCTransform method. Harmony was employed for batch correction and integration of datasets from different datasets and different tissue types while preserving biological variation. Principal component analysis (PCA) was performed on the integrated data, and uniform manifold approximation and projection (UMAP) was generated for visualisation. Unsupervised clustering was performed using the Louvain algorithm with a resolution parameter of 0.5.

### 2.2 Cell Type Annotation

Cell types within the tumour microenvironment were comprehensively annotated based on established lineage‐specific transcriptional signatures. We employed a panel of canonical marker genes to accurately classify diverse cellular populations. Epithelial cells were identified by EPCAM, KRT8 and KRT18 expression, while T lymphocytes were distinguished by T‐cell receptor components CD3D, CD3E and the cytotoxic marker CD8A. B lymphocytes were characterised through B‐cell receptor–associated CD79A and MS4A1. Natural killer cells expressed the cytotoxicity‐related markers KLRD1 and NCAM1. Myeloid lineage cells, including monocytes and macrophages, were identified by lysozyme (LYZ) and the scavenger receptor CD68. The stromal compartment was represented by fibroblasts expressing matrix proteins COL1A1 and decorin (DCN), while vascular endothelial cells were distinguished by PECAM1 and VWF expression.

### 2.3 Differential Gene Expression Analysis

Differential gene expression analysis between primary tumours and metastases was performed using the Wilcoxon rank‐sum test implemented in Seurat. Genes with an adjusted *p*‐value < 0.05 and absolute log2 fold change > 1 were considered differentially expressed.

### 2.4 Enrichment Analysis

Differentially expressed genes were then as input to perform KEGG enrichment and Gene set enrichment analysis (GSEA) using the clusterProfiler package in R with KEGG pathways [19]. Pathways with an adjusted *p*‐value < 0.05 were considered significantly enriched.

### 2.5 Cell–Cell Intracellular Communication Analysis

Intercellular communication was analysed using CellChat v2, a cutting‐edge computational framework that systematically infers and quantifies cell–cell interactions from single‐cell transcriptomic data [20]. We implemented a rigorous analytical pipeline that first normalised expression data from each metastatic site independently, followed by identifying overexpressed ligands and receptors in specific cell types. Communication probabilities were then computed based on the expression of cognate ligand–receptor pairs in sender and receiver cells, respectively, incorporating the law of mass action to model interaction strength. This approach enabled us to distinguish biologically meaningful communication patterns from random associations in the complex metastatic microenvironment. We focused our analysis on interactions between immune cells (particularly myeloid cells, T cells, B cells and NK cells) and epithelial cancer cells to identify key signalling axes that might drive metastatic colonisation in liver and lymph node metastases.

### 2.6 Cell Lines and Culture Conditions

Human gastric cancer cell lines were carefully selected to represent diverse molecular subtypes. GES‐1 (normal gastric epithelium), MKN‐45, MKN‐74, AGS and NCI‐N87 were obtained from the American Type Culture Collection (ATCC) with authentication certification. All cell lines were cultured in RPMI‐1640 medium supplemented with 10% heat‐inactivated fetal bovine serum and 1% penicillin–streptomycin solution (10,000 U/mL). Cultures were maintained at 37°C in a humidified incubator with a 5% CO_2_ atmosphere and routinely tested for mycoplasma contamination using PCR‐based methods.

### 2.7 Recombinant LGALS9 Treatment and Conditioned Medium Preparation

Recombinant human LGALS9 protein (R&D Systems) was added to the culture medium at a concentration of 150 ng/mL. For the preparation of LGALS9 conditioned medium, THP‐1 cells were differentiated into macrophages using PMA (100 nM) for 24 h, then transfected with the LGALS9 overexpression plasmid (pCMV‐LGALS9) or the control plasmid using Lipofectamine 3000 (Invitrogen). After 48 h, the culture medium was collected, centrifuged at 3000 rpm for 10 min to remove cellular debris and filtered through a 0.22 μm filter.

### 2.8 Pharmacological Inhibition of P4HB

Pharmacological inhibition of P4HB was achieved using two structurally distinct inhibitors to ensure target specificity and validate observed phenotypes through complementary approaches. Pharmacological inhibition of P4HB was achieved using quercetin‐3‐rutinoside (Q3R) (Sigma‐Aldrich) at 50 μM or bacitracin (BAC) (Sigma‐Aldrich) at 100 μM for the indicated time periods. Both compounds were dissolved in DMSO (final concentration < 0.1% in culture medium) and applied to cells for the time periods specified in individual experiments. Control conditions were treated with equivalent volumes of vehicle solutions to account for potential solvent effects.

### 2.9 Quantitative Real‐Time PCR

Total RNA was extracted using TRIzol reagent (Invitrogen) and reverse transcribed using the PrimeScript RT reagent Kit (Takara). Quantitative real‐time PCR (qRT‐PCR) was performed using SYBR Green PCR Master Mix (Applied Biosystems) on an ABI 7500 Real‐Time PCR System. GAPDH was used as an internal control, and relative gene expression was calculated using the 2‐ΔΔCt method.

### 2.10 Cell Viability Assay

Cell viability was assessed using the MTT assay. Briefly, cells were seeded in 96‐well plates (3000 cells/well) and treated as indicated. At specified time points, MTT solution (5 mg/mL) was added to each well and incubated for 4 h at 37°C. The formazan crystals were dissolved in DMSO, and absorbance was measured at 570 nm using a microplate reader.

### 2.11 EdU Incorporation Assay

Cell proliferation was evaluated using the Click‐iT EdU Alexa Fluor 647 Imaging Kit (Invitrogen) according to the manufacturer's instructions. Briefly, cells were incubated with 10 μM EdU for 2 h, fixed, permeabilised and stained with Alexa Fluor 647 azide and DAPI. Images were acquired using a fluorescence microscope (Olympus IX71) and analysed with ImageJ software.

### 2.12 Western Blot Analysis

Protein lysates were prepared using RIPA buffer containing protease inhibitors. Equal amounts of protein were separated by SDS‐PAGE and transferred to PVDF membranes. After blocking, membranes were incubated with primary antibodies against E‐cadherin, N‐cadherin, Vimentin and GAPDH (Cell Signalling Technology) overnight at 4°C, followed by incubation with HRP‐conjugated secondary antibodies. Protein bands were visualised using ECL detection reagent (Thermo Fisher Scientific).

### 2.13 Statistical Analysis

Statistical analyses were performed using R software (version 4.1.0). Data are presented as mean ± standard deviation (SD) from at least three independent experiments. Statistical significance was determined using Student's t‐test for two‐group comparisons or one‐way ANOVA followed by Tukey's post hoc test for multiple‐group comparisons. *p*‐values < 0.05 were considered statistically significant, with significance levels indicated as *p < 0.05, **p < 0.01 and ***p < 0.001.

## 3 Results

### 3.1 Single‐Cell Profiling Reveals Distinct Cellular Compositions in Primary Gastric Cancer and Metastatic Sites

**FIGURE 1:**
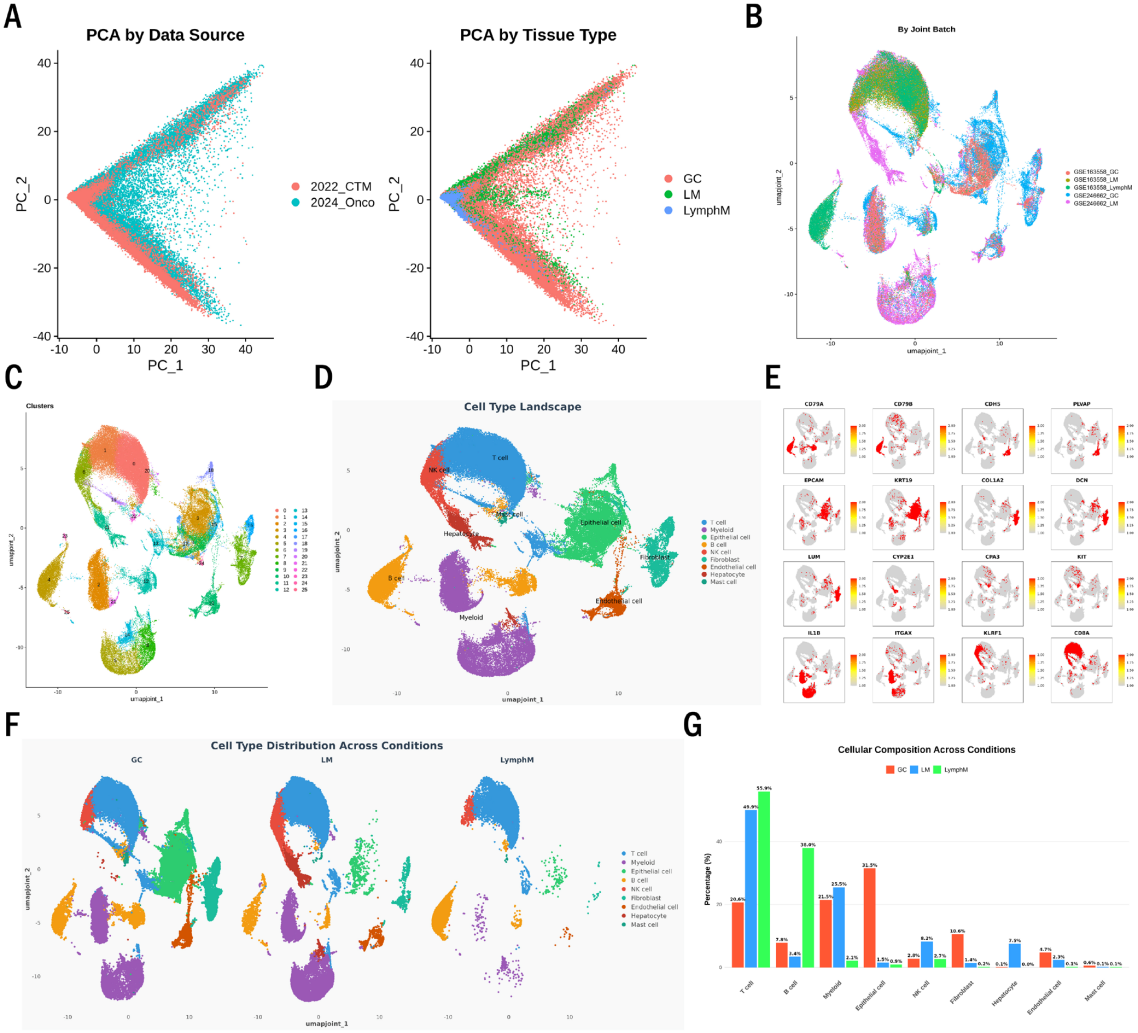
Single‐cell landscape of gastric cancer primary tumours and metastases. (A) Principal component analysis (PCA) coloured by data source and tissue type (right, GC, Gastric Cancer; LM, Liver Metastasis; LymphM, Lymph node Metastasis). (B) UMAP visualisation of integrated data from all samples coloured by dataset. (C) Unsupervised clustering of cells represented on UMAP. (D) Cell type annotation displayed on UMAP. (E) Expression of cell type–specific marker genes across the integrated dataset. (F) Cell type distribution across different conditions (GC, LM, LymphM) visualised on UMAP. (G) Cellular composition across different conditions shown as percentage of each cell type.

To comprehensively characterise the cellular composition and molecular features of gastric cancer metastasis, we performed an integrated analysis of scRNA‐seq data from primary gastric tumours (GC), liver metastases (LM) and lymph node metastases (LymphM). Our analysis incorporated two independent datasets (GSE246662 and GSE163558), comprising a total of 92,225 cells (43,720 GC, 36,419 LM and 12,086 LymphM cells) [10, 11]. Specifically, the GSE246662 dataset comprised 3 liver metastases samples paired with their 3 matched primary gastric cancer samples, while GSE163558 contributed an additional 3 primary tumour samples, 2 liver metastasis samples and 2 lymph node metastasis samples. This cohort, though limited in size (6 primary tumours, 5 liver metastases and 2 lymph node metastases), provides valuable initial insights into metastatic progression. Principal component analysis (PCA) revealed distinct clustering patterns based on data source and tissue type (Figure 1A), reflecting both batch effects and biological variation. After integration and batch correction, cells displayed a more homogeneous distribution in the UMAP representation (Figure 1B), enabling unbiased comparison across different tissue sites. Unsupervised clustering identified 26 distinct cell clusters (Figure 1C), which were annotated based on the expression of canonical marker genes as epithelial cells, T cells, B cells, NK cells, myeloid cells, fibroblasts and endothelial cells (Figure 1D,E).

Comparison of cellular compositions across the three sites revealed significant differences in immune cell proportions (Figure 1F,G). Notably, T cells constituted a higher percentage in LymphM (55.9%) compared to GC (20.6%) and LM (49.9%), whereas myeloid cells were more abundant in GC (21.5%) and LM (25.5%) than in LymphM (2.1%). Epithelial cells were also more abundant in GC (31.5%) than in LM (1.5%) and LymphM (0.9%). These results suggest that immune cells play important roles in gastric cancer metastasis.

### 3.2 Metastatic Gastric Cancer Cells Exhibit Distinct Transcriptional Programs With Enhanced Lipid Metabolism

**FIGURE 2:**
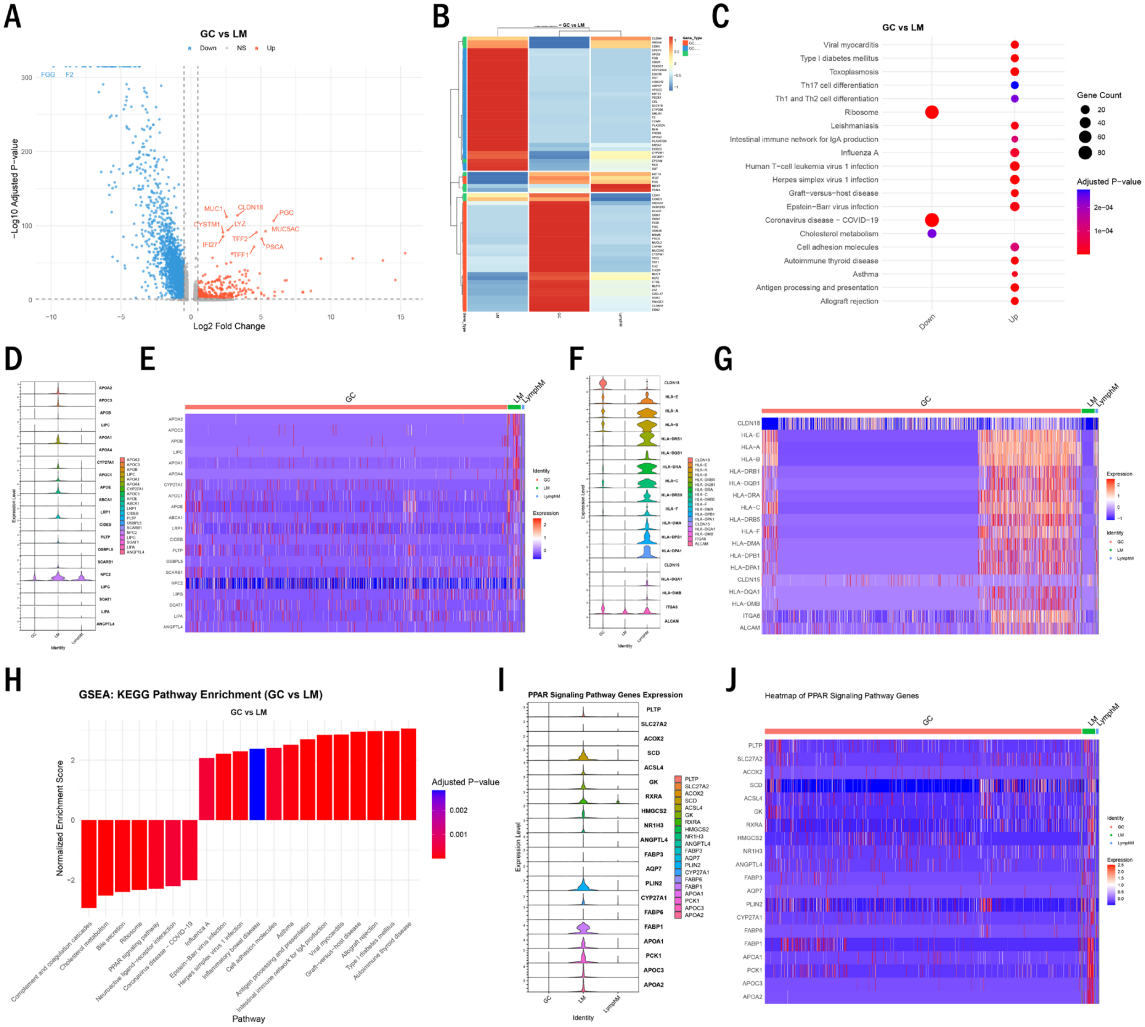
Metastatic gastric cancer cells exhibit upregulated lipid metabolism pathways. (A) Volcano plot showing differentially expressed genes between epithelial cells from GC and LM. Red dots represent significantly upregulated genes in LM, blue dots represent significantly downregulated genes. (B) Heatmap showing expression patterns of top differentially expressed genes among GC, LM and LymphM epithelial cells. (C) Dot plot showing enriched KEGG pathways in differentially expressed genes between GC and LM epithelial cells. (D) Violin plots showing expression of representative cholesterol metabolism genes in epithelial cells from GC, LM and LymphM (E) Heatmap showing expression of cholesterol metabolism‐related genes in epithelial cells from GC, LM and LymphM. (F) Violin plots showing expression of representative Th17 cell differentiation pathway genes. (G) Heatmap showing expression of Th17 cell differentiation–related genes. (H) GSEA enrichment plot showing significantly enriched KEGG pathways in LM compared to GC epithelial cells. (I) Violin plots showing expression of PPAR signalling pathway genes. (J) Heatmap showing expression of PPAR signalling pathway genes.

To identify molecular alterations between primary gastric cancer site and metastasis site, we performed differential gene expression analysis comparing epithelial cells from primary tumours and metastases. The volcano plot revealed 2982 differentially expressed genes (DEGs) between GC and LM epithelial cells (Figure 2A), with 694 upregulated and 2288 downregulated genes in LM. Hierarchical clustering of the top DEGs revealed distinct expression patterns between GC and LM epithelial cells (Figure 2B). Functional enrichment analysis of DEGs revealed significant enrichment of cholesterol metabolism pathways in LM epithelial cells, while Th17 cell differentiation pathways were downregulated (Figure 2C). Violin plots and heatmap visualisation demonstrated elevated expression of cholesterol metabolism genes, including NPC2, APOA and SCD, in LM compared to GC epithelial cells, suggesting altered metabolism modulation in the metastatic microenvironment (Figure 2D,E). Conversely, genes associated with Th17 cell differentiation, including CLDN18 and ITGA6, showed reduced expression in LM epithelial cells (Figure 2F,G). Gene Set Enrichment Analysis (GSEA) confirmed the enrichment of cholesterol metabolism and additionally identified PPAR signalling pathway as significantly upregulated in LM epithelial cells (Figure 2H). Expression patterns of PPAR signalling genes, including FABP1 and PLIN2, were consistently elevated in metastatic cells (Figure 2I,J).

### 3.3 Cell–Cell Communication Analysis Reveals LGALS9–P4HB Interaction as a Key Mediator of Myeloid–Epithelial Cell Crosstalk in Metastatic Sites

**FIGURE 3:**
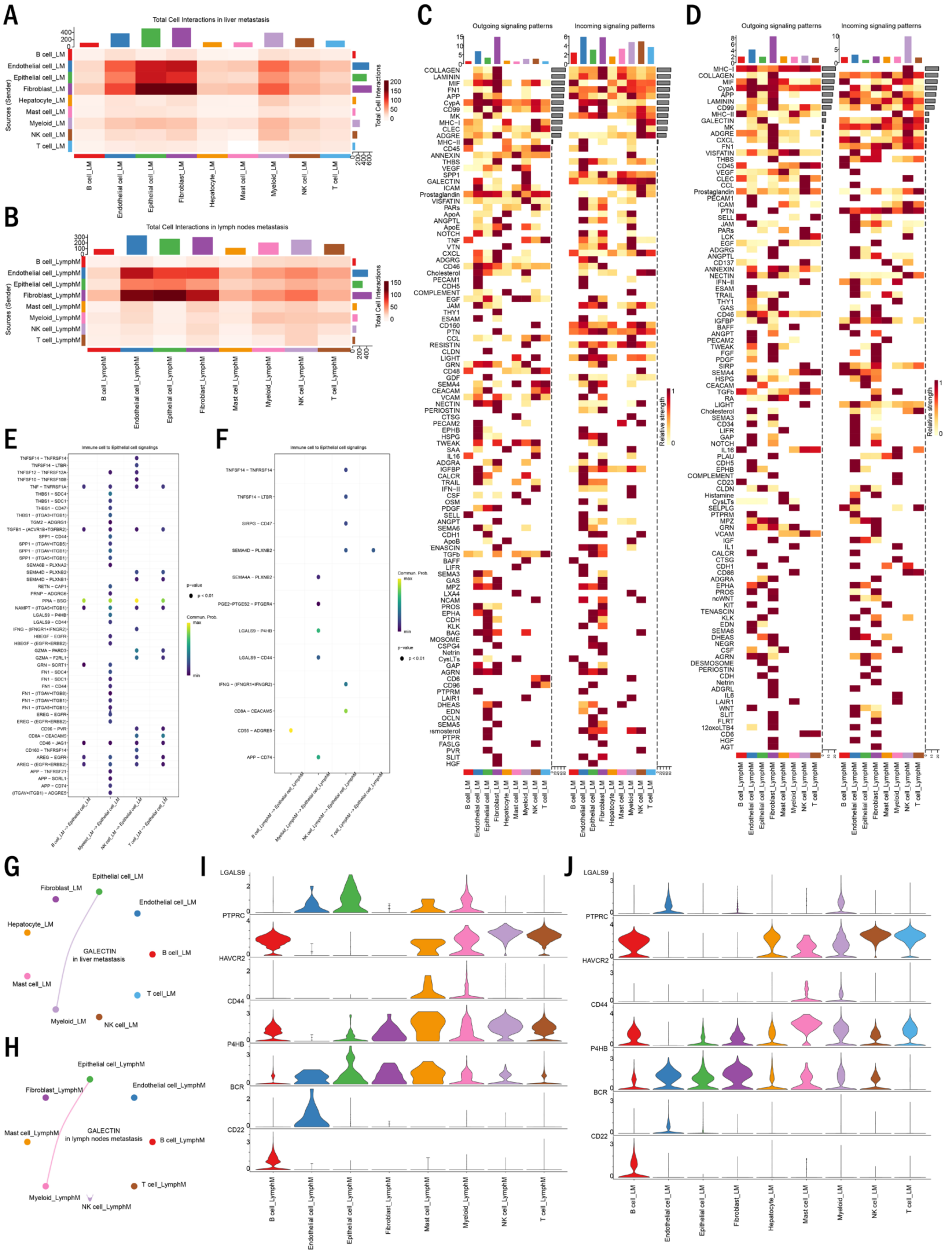
Cell–cell communication analysis reveals LGALS9–P4HB as a key interaction between myeloid and epithelial cells in metastatic sites. (A) Heatmap showing total cell interactions in liver metastases. (B) Heatmap showing total cell interactions in lymph node metastases. (C) Outgoing and incoming signalling patterns in liver metastases. (D) Outgoing and incoming signalling patterns in lymph node metastases. (E) Dot plot showing ligand–receptor pairs mediating immune cell–epithelial cell communication in liver metastases. (F) Dot plot showing ligand–receptor pairs mediating immune cell–epithelial cell communication in lymph node metastases. (G) Network diagram of cellular interactions in liver metastases. (H) Network diagram of cellular interactions in lymph node metastases. (I) Violin plots showing expression of LGALS9 and its receptors (P4HB, HAVCR2, CD44) across different cell types in liver metastases. (J) Violin plots showing expression of LGALS9 and its receptors across different cell types in lymph node metastases.

To elucidate the intercellular signalling networks underlying metastatic colonisation, especially focusing on the interactions between immune cells and epithelial cells, we performed cell–cell communication analysis using CellChat [20]. The analysis of total cellular interactions in liver and lymph node metastases revealed complex communication patterns among different cell types (Figure 3A,B). Notably, endothelial cells, epithelial cells, fibroblasts and myeloids exhibited the highest number of outgoing and incoming signals to epithelial cells in both metastatic sites. Detailed analysis of signalling pathways and their cellular sources and targets identified distinct communication patterns within the metastatic microenvironment (Figure 3C,D). We focused on ligand–receptor pairs mediating immune cell–epithelial cell interactions and identified several conserved pairs present in both metastatic sites (Figure 3E,F). Among these, the LGALS9–P4HB interaction between myeloid cells and epithelial cells emerged as a significant communication axis (Figure 3G,H). Violin plot analysis confirmed the expression of LGALS9 in myeloid cells and P4HB in epithelial cells across both metastatic sites (Figure 3I,J), suggesting a conserved signalling mechanism in gastric cancer metastasis.

### 3.4 LGALS9 Activates P4HB to Promote Gastric Cancer Cell Proliferation, EMT and Lipid Metabolism

**FIGURE 4:**
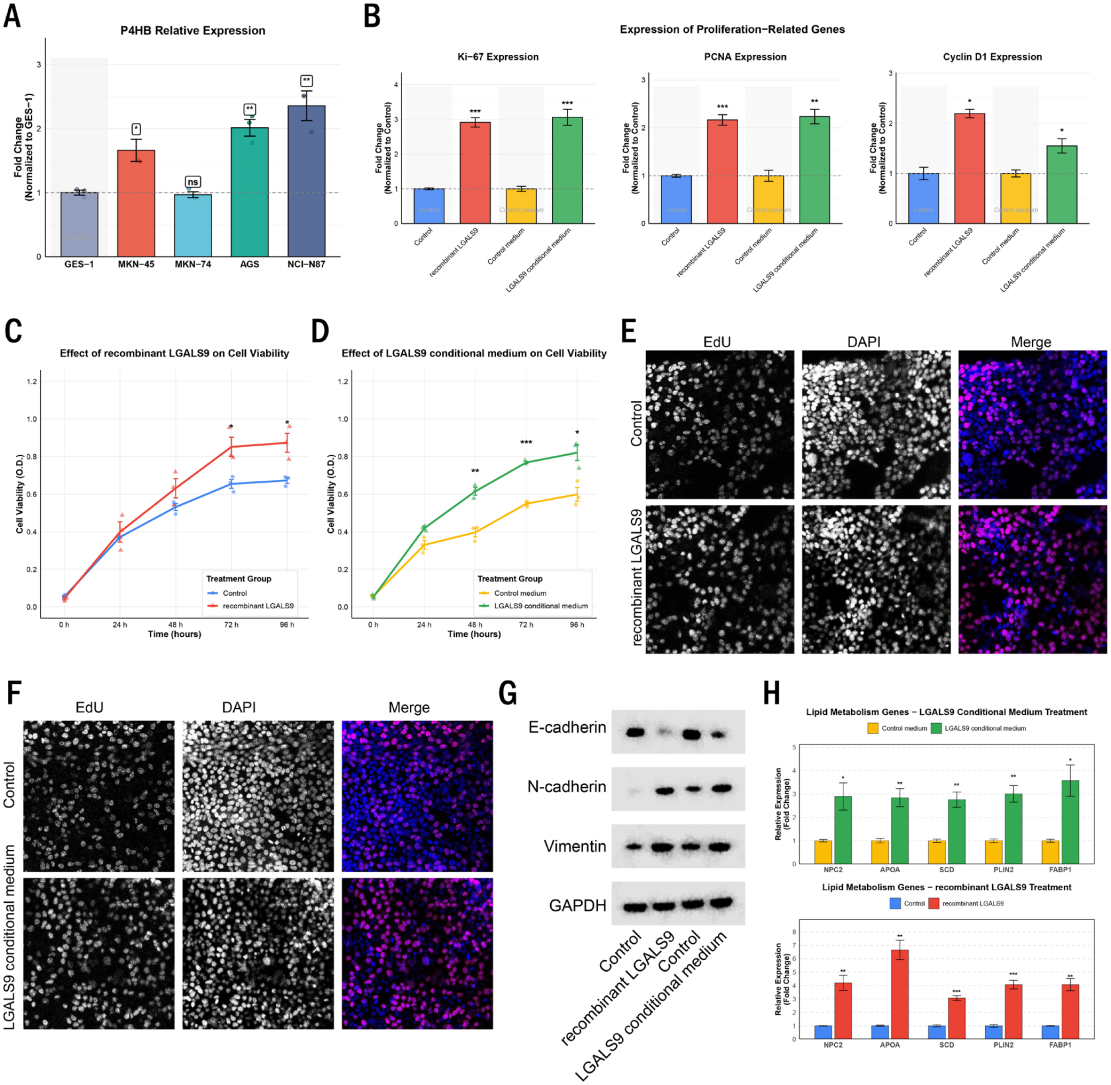
LGALS9 activates P4HB to promote gastric cancer cell proliferation, EMT and lipid metabolism. (A) Relative expression of P4HB in different gastric cell lines measured by qRT‐PCR. (B) Expression of proliferation‐related genes (Ki‐67, PCNA, Cyclin D1) in MKN‐74 cells treated with recombinant LGALS9 or LGALS9 conditional medium, determined by qRT‐PCR. (C) Cell viability of MKN‐74 cells treated with recombinant LGALS9 over time, measured by MTT assay. (D) Cell viability of MKN‐74 cells cultured in LGALS9 conditional medium over time. (E) EdU incorporation assay showing proliferation of MKN‐74 cells treated with recombinant LGALS9. (F) EdU incorporation assay showing proliferation of MKN‐74 cells cultured in LGALS9 conditional medium. (G) Western blot analysis of EMT markers (E‐cadherin, N‐cadherin, Vimentin) in MKN‐74 cells with indicated treatments. (H) qRT‐PCR analysis of lipid metabolism–related genes in MKN‐74 cells treated with LGALS9 conditional medium (upper panel) or recombinant LGALS9 (lower panel). Statistical significance is indicated by asterisks: *p < 0.05, **p < 0.01, ***p < 0.001, ns: Not significant.

To validate the functional relevance of the LGALS9–P4HB interaction, we first examined P4HB expression across normal gastric cell line GES‐1 and various gastric cancer cell lines. qRT‐PCR analysis revealed differential P4HB expression levels, with MKN‐45 and MKN‐74 showing relatively low expression, while NCI‐N87 exhibited the highest expression compared to the normal gastric epithelial cell line GES‐1 (Figure 4A). Based on these expression profiles, we selected MKN‐74 (low P4HB expression) for gain‐of‐function studies using recombinant LGALS9 protein or LGALS9 conditioned medium. Treatment with either recombinant LGALS9 or LGALS9 conditioned medium significantly increased the expression of proliferation markers Ki‐67, PCNA and Cyclin D1 in MKN‐74 cells (Figure 4B). Cell viability assays demonstrated enhanced proliferation of MKN‐74 cells upon treatment with recombinant LGALS9 or LGALS9 conditioned medium over a 96‐h period (Figure 4C,D). This proliferative effect was further confirmed by EdU incorporation assays, which showed increased DNA synthesis in treated cells (Figure 4E,F). Western blot analysis revealed that LGALS9 treatment promoted epithelial–mesenchymal transition (EMT) in MKN‐74 cells, as evidenced by decreased E‐cadherin and increased N‐cadherin and Vimentin expression (Figure 4G). Consistent with our transcriptomic findings, LGALS9 treatment upregulated the expression of genes involved in cholesterol metabolism (NPC2, APOA) and PPAR signalling (SCD, PLIN2, FABP1) (Figure 4H), suggesting that LGALS9‐P4HB interaction modulates lipid metabolism in gastric cancer cells.

### 3.5 Pharmacological Inhibition of P4HB Suppresses Gastric Cancer Cell Proliferation, EMT and Lipid Metabolism

**FIGURE 5:**
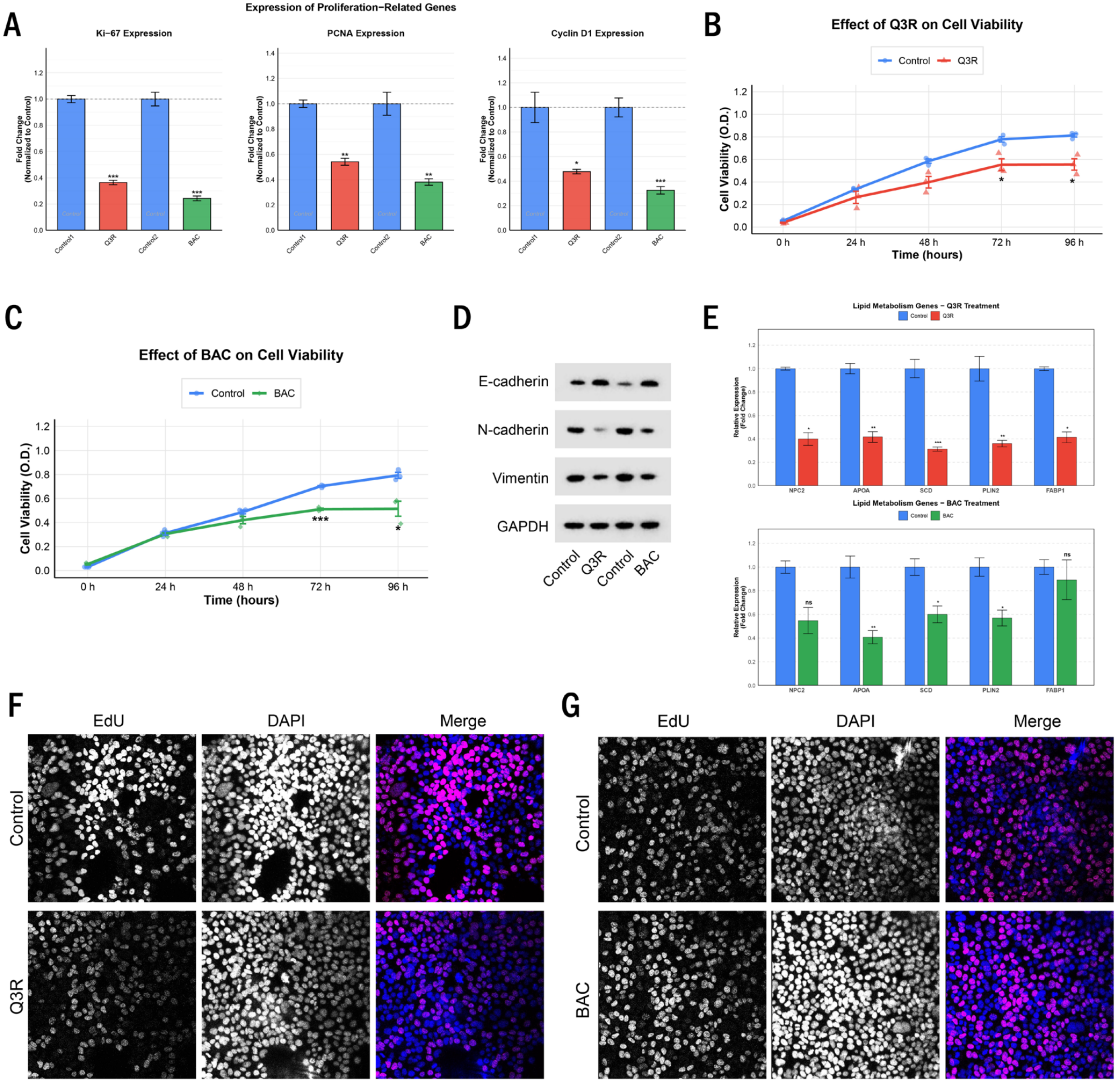
Pharmacological inhibition of P4HB suppresses gastric cancer cell proliferation, EMT and lipid metabolism. (A) Expression of proliferation‐related genes (Ki‐67, PCNA, Cyclin D1) in NCI‐N87 cells treated with P4HB inhibitors (Q3R or BAC), determined by qRT‐PCR. (B) Cell viability of NCI‐N87 cells treated with Q3R over time, measured by MTT assay. (C) Cell viability of NCI‐N87 cells treated with BAC over time. (D) Western blot analysis of EMT markers in NCI‐N87 cells with indicated treatments. (E) qRT‐PCR analysis of lipid metabolism–related genes in NCI‐N87 cells treated with Q3R (upper panel) or BAC (lower panel). (F) EdU incorporation assay showing proliferation of NCI‐N87 cells treated with Q3R. (G) EdU incorporation assay showing proliferation of NCI‐N87 cells treated with BAC. Statistical significance is indicated by asterisks: *p < 0.05, **p < 0.01, ***p < 0.001, ns: Not significant.

To further validate the role of P4HB in mediating the effects of LGALS9, we employed pharmacological inhibitors of P4HB (quercetin‐3‐rutinoside [Q3R] and bacitracin [BAC]) in NCI‐N87 cells, which express high levels of P4HB. Treatment with either Q3R or BAC significantly reduced the expression of proliferation markers in NCI‐N87 cells (Figure 5A), consistent with a decrease in cell viability over time (Figure 5B,C). EdU incorporation assays confirmed the anti‐proliferative effects of P4HB inhibition (Figure 5F,G). Western blot analysis demonstrated that P4HB inhibition reversed the EMT phenotype, as evidenced by increased E‐cadherin and decreased N‐cadherin and Vimentin expression (Figure 5D). Furthermore, P4HB inhibition downregulated the expression of cholesterol metabolism and PPAR signalling genes in NCI‐N87 cells (Figure 5E), supporting the role of P4HB in regulating lipid metabolism in gastric cancer cells. These results indicate that P4HB is necessary for gastric cancer cell proliferation, EMT and the upregulation of lipid metabolism.

## 4 Discussion

Metastasis remains the primary cause of mortality in gastric cancer patients; however, the molecular mechanisms underlying this process remain incompletely understood. In this study, we integrated single‐cell transcriptomic analysis of primary gastric tumours and matched metastases with functional experiments to identify a novel interaction between myeloid cell‐derived LGALS9 and epithelial cell‐expressed P4HB that promotes metastatic colonisation through enhanced proliferation, EMT and altered lipid metabolism.

Our comprehensive cellular landscape of gastric cancer metastasis revealed distinct immune compositions in different metastatic sites, with a higher proportion of T cells in lymph node metastases and more abundant myeloid cells in liver metastases. These findings align with previous reports of tissue‐specific immune microenvironments in various metastatic sites [21, 22]. The observed differences in immune cell proportions likely reflect both the native immune composition of the respective organs and the specific immune responses elicited by metastatic colonisation.

Transcriptomic comparison of epithelial cells from primary tumours and liver metastases revealed significant upregulation of cholesterol metabolism and PPAR signalling pathway genes in metastatic cells. This finding is consistent with emerging evidence suggesting that altered lipid metabolism plays a crucial role in metastatic progression [23, 24]. Enhanced cholesterol metabolism may provide metastatic cells with the necessary building blocks for membrane synthesis during rapid proliferation or confer survival advantages in the challenging microenvironment of distant organs [25, 26, 27].

Our cell–cell communication analysis identified LGALS9‐P4HB as a key interaction between myeloid cells and epithelial cells in metastatic sites. Galectin‐9 (LGALS9), a β‐galactoside‐binding lectin, has been implicated in various aspects of cancer biology, including immune modulation and metastasis. P4HB, primarily known for its role in protein folding in the endoplasmic reticulum, has recently been recognised as a cell surface receptor involved in cellular adhesion and migration. Our study is the first to identify P4HB as a functional receptor for LGALS9 in gastric cancer, establishing a novel signalling axis in the metastatic microenvironment. Our identification of the myeloid‐derived LGALS9‐P4HB signalling axis represents a significant advancement in understanding tumour–immune interactions within the metastatic microenvironment. Rather than serving as passive inflammatory mediators, myeloid cells actively instruct epithelial cell behaviour through metabolic reprogramming, proliferation enhancement and EMT induction. This immune–epithelial crosstalk likely contributes to organ‐specific metastatic patterns in gastric cancer and suggests that therapeutic strategies should target both tumour cells and their supporting immune signals. Disrupting this communication network could potentially sensitise metastatic cells to conventional therapies by removing critical extrinsic support mechanisms from the immune microenvironment.

Functional experiments demonstrated that LGALS9 activation of P4HB promotes gastric cancer cell proliferation, EMT and expression of lipid metabolism genes. These findings suggest that the LGALS9–P4HB interaction may serve as a mechanism by which myeloid cells in the metastatic microenvironment support the colonisation and growth of disseminated tumour cells. The observed effects on EMT are particularly notable, as this process has been implicated in metastatic initiation, colonisation and therapy resistance [28, 29, 30, 31]. The upregulation of lipid metabolism genes following LGALS9 treatment establishes a mechanistic link between myeloid‐epithelial cell communication and the metabolic reprogramming observed in metastatic cells. This connection highlights the complex interplay between the tumour microenvironment and cancer cell metabolism in driving metastatic progression.

Pharmacological inhibition of P4HB using Q3R or BAC effectively reversed the pro‐metastatic effects of LGALS9, suppressing proliferation, EMT and lipid metabolism in gastric cancer cells. Q3R (quercetin‐3‐rutinoside) demonstrates notable specificity for P4HB among the protein disulfide isomerase family. Experimental studies show that Q3R effectively inhibits P4HB activity, particularly by blocking conformational changes essential for its catalytic function, representing a flavonoid derivative that exhibits high binding affinity for the b’ domain of P4HB with an IC50 of approximately 6.1 μM [32, 33, 34]. Bacitracin, a structurally unrelated cyclic polypeptide antibiotic, inhibits P4HB through an entirely different mechanism by forming disulfide bonds with free cysteines in the substrate‐binding domain of the enzyme [35]. This differential inhibitory mechanism provides a critical orthogonal validation of our Q3R findings. The convergent phenotypes observed with both inhibitors, despite their distinct chemical structures and inhibitory mechanisms, strongly support P4HB as the specific mediator of the observed effects rather than off‐target activities. These findings suggest that targeting the LGALS9–P4HB interaction or downstream signalling pathways may represent a novel therapeutic strategy for metastatic gastric cancer. Given the poor prognosis and limited treatment options for metastatic gastric cancer, the identification of new therapeutic targets is of paramount importance [36, 37, 38].

Several limitations of our study should be acknowledged. First, while our integrated analysis included primary tumours and matched metastases, the sample size was relatively limited. This limitation particularly affects our ability to account for potentially confounding variables such as tumour subtype heterogeneity, treatment history and patient‐specific genetic backgrounds. Future investigations employing substantially expanded cohorts—ideally incorporating longitudinal sampling and diverse ethnic populations—will be instrumental in validating our observations and potentially uncovering additional metastatic mechanisms that may have eluded detection in our current dataset. Moreover, integration with spatial transcriptomics and functional validation studies would significantly strengthen the clinical applicability of the metastatic signatures we have identified, potentially revealing therapeutic vulnerabilities unique to specific metastatic niches. Second, while our current study demonstrates a clear association between LGALS9–P4HB interaction and upregulation of lipid metabolism genes, we acknowledge that the precise molecular mechanisms mediating this relationship require further elucidation. P4HB (protein disulfide isomerase A1) primarily functions as an endoplasmic reticulum (ER) chaperone involved in protein folding. However, when expressed on the cell surface, it can act as a signalling receptor that potentially activates multiple downstream pathways. P4HB has been shown to influence EMT and cancer cell invasiveness, both of which are processes frequently regulated by PI3K/AKT signalling [39, 40, 41]. We hypothesize that LGALS9 binding to cell surface P4HB may trigger conformational changes that activate this pathway, ultimately leading to transcriptional upregulation of lipid metabolism genes. Alternatively, P4HB activation might influence ER stress responses [42, 43], which have been implicated in metabolic reprogramming during cancer progression. LGALS9–P4HB interaction could potentially modulate these ER stress pathways, providing another mechanism for the observed metabolic alterations. In our future studies, we plan to systematically investigate these potential mechanisms using pharmacological inhibitors, genetic knockdown approaches and biochemical assays to establish the direct molecular link between P4HB activation and lipid metabolism dysregulation. These investigations will likely reveal novel therapeutic opportunities for targeting metabolic vulnerabilities in metastatic gastric cancer. Third, although we demonstrated the functional relevance of the LGALS9–P4HB interaction in vitro, in vivo validation using animal models would strengthen our findings. Specifically, orthotopic xenograft models with LGALS9 overexpression or P4HB knockdown would provide valuable insights into how this interaction influences metastatic dissemination and colonisation in physiologically relevant microenvironments. Furthermore, patient‐derived xenografts treated with pharmacological inhibitors of P4HB would offer translational evidence for the therapeutic potential of targeting this pathway.

In conclusion, our study establishes LGALS9–P4HB interaction as a critical mediator of gastric cancer metastatic colonisation through enhanced proliferation, EMT and altered lipid metabolism. These findings provide insights into the complex intercellular communication networks within the metastatic microenvironment and identify potential therapeutic targets for metastatic gastric cancer.

## Author Contributions

**Xiaobin Zhu:** conceptualization (equal), data curation (equal), writing – original draft (supporting), writing – review and editing (supporting). **Yating Zhang:** conceptualization (equal), formal analysis (equal), investigation (equal), methodology (equal), writing – original draft (supporting), writing – review and editing (supporting). **Aiping Yu:** formal analysis (equal), investigation (equal), methodology (equal), writing – review and editing (supporting). **Xiao Xiao:** resources (lead), supervision (lead), writing – original draft (lead), writing – review and editing (lead).

## Conflicts of Interest

The authors declare no conflicts of interest.

## Data Availability

The single‐cell RNA sequencing data used in this study are available from the Gene Expression Omnibus (GEO) under accession numbers GSE246662 and GSE163558.
